# Retraction: Fast fault diagnosis of smart grid equipment based on deep neural network model based on knowledge graph

**DOI:** 10.1371/journal.pone.0345825

**Published:** 2026-04-08

**Authors:** The PLOS One Editors

The *PLOS One* Editors retract this article [1] due to concerns about potential manipulation of the publication process. These concerns call into question the validity and provenance of the reported results. We regret that the issues were not identified prior to the article’s publication.

All authors either did not respond directly or could not be reached.

## References

[1] Jun L, Chenliang Z. RETRACTED: Fast fault diagnosis of smart grid equipment based on deep neural network model based on knowledge graph. PLoS One. 2025;20(2):e0315143. doi: 10.1371/journal.pone.0315143 39951439 PMC11828369

